# Phosphorous Diffuser Diverged Blue Laser Diode for Indoor Lighting and Communication

**DOI:** 10.1038/srep18690

**Published:** 2015-12-21

**Authors:** Yu-Chieh Chi, Dan-Hua Hsieh, Chung-Yu Lin, Hsiang-Yu Chen, Chia-Yen Huang, Jr-Hau He, Boon Ooi, Steven P. DenBaars, Shuji Nakamura, Hao-Chung Kuo, Gong-Ru Lin

**Affiliations:** 1 Graduate Institute of Photonics and Optoelectronics, National Taiwan University (NTU), No. 1, Sec. 4, Roosevelt Road, Taipei 10617, Taiwan, Republic of China; 2 Department of Photonics, National Chiao Tung University, No. 1001 Ta Hsueh Rd., Hsinchu 30050, Taiwan, Republic of China; 3 Computer, Electrical and Mathematical Sciences and Engineering (CEMSE) division, King Abdullah University of Science & Technology (KAUST), Thuwal 23955-6900, Saudi Arabia; 4 Materials Department, University of California, Santa Barbara, CA 93106, USA; 5 Electrical and Computer Engineering Department, University of California, Santa Barbara, CA 93106, USA; 6 Department of Electrical Engineering, National Taiwan University (NTU), No. 1, Sec. 4, Roosevelt Road, Taipei 10617, Taiwan, Republic of China

## Abstract

An advanced light-fidelity (Li-Fi) system based on the blue Gallium nitride (GaN) laser diode (LD) with a compact white-light phosphorous diffuser is demonstrated for fusing the indoor white-lighting and visible light communication (VLC). The phosphorous diffuser adhered blue GaN LD broadens luminescent spectrum and diverges beam spot to provide ample functionality including the completeness of Li-Fi feature and the quality of white-lighting. The phosphorous diffuser diverged white-light spot covers a radiant angle up to 120^o^ with CIE coordinates of (0.34, 0.37). On the other hand, the degradation on throughput frequency response of the blue LD is mainly attributed to the self-feedback caused by the reflection from the phosphor-air interface. It represents the current state-of-the-art performance on carrying 5.2-Gbit/s orthogonal frequency-division multiplexed 16-quadrature-amplitude modulation (16-QAM OFDM) data with a bit error rate (BER) of 3.1 × 10^−3^ over a 60-cm free-space link. This work aims to explore the plausibility of the phosphorous diffuser diverged blue GaN LD for future hybrid white-lighting and VLC systems.

Light-fidelity (Li-Fi) for both indoor luminescent white-lighting and optical wireless communication (or visible light communication, VLC) has recently been a research spotlight because of its advantages of license-free, high secrecy, non-electromagnetic interference and potential integration with current luminaire^1^,^2^,^3^,^4^,^5^,^6^. Originally, the concept of VLC was conceived in Japan^7^,^8^, and the increasing interests are booming up to now. Remarkably, Minh *et al.* used 16 white LEDs to combine with a multiple-resonant equalization technique for implementing the 40-Mbit/s VLC in 2008^9^. Later on, similar VLC with 100-Mbit/s nonreturn-to-zero (NRZ) format using a post-equalized white LED was demonstrated by the same group^10^. In the same year, Vucic *et al.* preliminarily used a thin-film high-power phosphorescent white-light LED and a discrete-multitone modulation to demonstrate a 200+ Mbit/s VLC^11^, and further upgraded its transmission capacity to 513 Mbit/s in 2010^1^. Khalid *et al.*^12^ and Wu *et al.*^13^ respectively employed the rate-adaptive discrete multitone modulation and the carrier-less amplitude and phase (CAP) modulation, enabling the commercial phosphorescent white-light LED based VLC system beyond 1 Gbit/s as late as 2012. In a future prospection, the schematic diagram of Li-Fi system using a white-light source for not only lighting but also communication is illustrated in Fig. 1, in which all electrical equipments and components in house can transmit and receive data *via* general illumination with Li-Fi functionality, and the emerging techniques such as optical multiple input multiple output (MIMO) will be considered to further achieve high data rates^14^.

In view of previous works, either mixing red/green/blue (RGB) light emitting diodes (LEDs), or combining a blue LED with a yellow phosphor can be considered as an alternative approach for white-light generation^15^,^16^,^17^,^18^,^19^. In 2013, Wang *et al.* used RGB LEDs and phosphor-based LED to demonstrate subcarrier multiplexed and wavelength division multiplexed (SCM-WDM) bi-directional VLC system with 575-Mbit/s downlink and 225-Mbit/s uplink, respectively^20^. More recently, the WDM VLC systems based on a CAP modulated RGB LED at 3.22 and 4.5 Gbit/s were consecutively proposed by Wu *et al.* in 2013^18^ and Wang *et al.* in 2015^21^. When comparing the white light source by mixing RGB LEDs, the blue LED illuminated yellow phosphor based lighting element is more compact and cost-effective; however, it suffers from a reduced data rate because the passively excited yellow phosphor is only a spontaneous emission source with lifetime much longer than the LED, which could degrade the transmitted data carried by the blue light. Fortunately, this problem can be alleviated with new class of data formats such as orthogonal frequency division multiplexed (OFDM) high-level quadrature amplitude modulation (QAM) based data with high spectral usage efficiency^22^,^23^.

In addition, the micro-LEDs with compact device architecture and large driving capacitance would be an alternative candidate to increase the data bandwidth^24^. In 2014, Chun *et al.* started to use the micro-LED combined with a yellow fluorescent copolymer to implement the fast white-light VLC at 1.68 Gbit/s^25^. Nevertheless, the efficiency droop phenomenon under high current injection level has practically limited the modulation and lighting performances of the micro-LED^26^,^27^,^28^,^29^,^30^. To release such a bottleneck, the integration of visible laser diodes (LDs) into the Li-Fi system is proposed for taking its advantages including broader modulation bandwidth and higher pumping efficiency than currently available LEDs^31^. To meet this demand, a 532-nm VLC at 1 Gbit/s through a 2-m water pipe was demonstrated by Hanson *et al.*^32^, and a red LD based bidirectional 2.5-Gbit/s VLC with 16-QAM OFDM over 20-km single-mode fiber (SMF) and 15-m free-space was reported by Chen *et al.*^33^. Singh *et al.* further proposed a directly-modulated 641-nm laser pointer based VLC with 4-QAM OFDM data^34^. To construct the LD based Li-Fi system, gallium nitride (GaN) blue LD is preliminarily considered as the most preferable device among all visible LDs^35^ because of its potential for both white-light illumination with ultrahigh brightness and data transmission with large bandwidth. In 2013, Wierer *et al.* have compared the economics and input-power-density dependent power-conversion efficiencies between blue LEDs and LDs based Li-Fi systems^36^. Moreover, the potential of III-nitride LD based lighting was also emerged by the same group, which declares the higher efficiency of blue LDs than that of blue LEDs at larger input power densities^37^. To implement the white-lighting system, a high-luminescent white-light source with a luminance as high as 85 cd/mm^2^ by combining a high-power blue LD, an optical fiber and a phosphor was developed by Kozaki *et al.*^38^. Alternatively, Hashimoto *et al.* fabricated a high-power 2.8-W blue-violet InGaN LD with AlN facet coating, and demonstrated the phosphor-converted LD excitation white-light source with luminous flux over 380 lm^39^. By combining four-color LDs and passing through a diffuser, Neumann *et al.* demonstrated a white illuminant^40^. In addition, the direct laser activation of a remote ceramic phosphor with high thermal conductivity for high luminous fluxes and luminance was reported by Lenef *et al.*^41^. For implementing the blue LD based VLC system, Watson *et al.* demonstrated a directly modulated 422-nm GaN LD based VLC at 2.5 Gbit/s^42^, and a 450-nm GaN LD based VLC with 9-Gbit/s QAM-OFDM over a 5-m free-space link was just released lately^43^. Tsonev *et al.*^44^ and Janjua *et al.*^45^ have individually used commercial RGB LDs for simultaneous white-light generation and optical data transmission with individual LD at data rate beyond 4 Gbit/s. Later on, Chun *et al.* combined a blue LD with a remote phosphor for white-lighting application, providing the highest data rate of up to 6.52 Gbit/s for free-space link over 15-cm^46^. Blue LD with adhered yellow phosphor is a potential solution to synthesis white light for laser lighting instead of using RGB lasers. Except for the result from previous work^46^, few researches were focused on the yellow phosphor encapsulated blue LD for VLC systems. Many issues in LD based lighting system have yet been solved to compete the existing LED lighting system. First, conventional yellow phosphor cannot be directly coated on the LD facet for generating white light due to heat dissipation issues. Second, the balance between lighting quality and data transmission needs to be proposed with potential approaches for augmenting the performance on both purposes.

In this work, a free standing phosphorous diffuser adhered blue LD based white-light source is proposed for both indoor white-lighting and optical wireless communication without direct contact on LDs. The flexible phosphor film can be easily attached onto versatile holders or lenses for secondary optics. It successfully delivers 16-QAM OFDM data at a raw data rate of up to 5.2 Gbit/s over a 60-cm free-space link, which exhibits an error vector magnitude (EVM) of 17.3%, a signal-to-noise ratio (SNR) of 15.3 dB and a bit error rate (BER) of 3.1 × 10^−3^. To the best of our knowledge, the present system provides the highest data rate while comparing with previous works at the same free-space distance. This work explores the transmission capability of proposed white-light source for next-generation Li-Fi application including phone-to-phone and vehicle-to-vehicle communications. The luminescent white-lighting performance of the phosphorous diffuser adhered blue LD is addressed in Section I of Results. In Section II of Results, the transmission performance of blue LD with yellow phosphorous diffuser carried 16-QAM OFDM data over a 60-cm free-space link is also performed.

## Results

### The luminescent white-lighting performance of the phosphorous diffuser adhered blue LD

**Figure 2:**
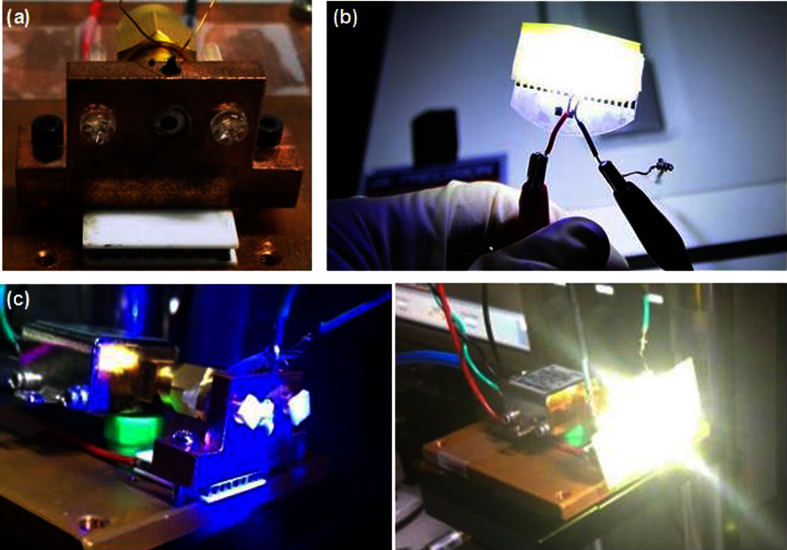
The temperature controlled TO38-can packaged blue LD with phosphorous diffuser for indoor white-lighting. (**a**) The TO38-can packaged blue LD with a homemade temperature controller. (**b**) The image of phosphor film excited by the blue laser beam. (**c**) The zoom-in image of the blue LD without and with phosphor.

A homemade temperature controller^43^ is added to stabilize the output dynamics of blue GaN LD (λ_emission_ = 450 nm) packaged with 38 transistor outline (TO38) can, as shown in Fig. 2(a). The phosphor used in this experiment is the commercially available Y_3_Al_5_O_12_:Ce^3+^ (YAG) powder with an emission peak wavelength of 550 nm and an average size of about 13 μm, which provides broadband luminescence and scattered diffusion to transfer the highly oriented blue laser beam into a widely divergent white-light distribution. Figure 2(b) displays the white-lighting result of the blue LD illuminating phosphor doped diffuser, showing bright and divergent white-light beam based on the combination of blue light and excited yellow light. With such a phosphor, the blue LD transfers its coherent blue light into brightly divergent white light, as shown in Fig. 2(c). Note that the luminous flux is 6.6 lm after propagating through the same distance for data transmission (60 cm), which is somewhat low for lighting purpose when comparing to the brightness of conventional white-light LEDs. High power LDs might solve this problem; however, most of commercially available high-power LDs choose to increase metal pad size for enlarging the active volume, which would suffer from large capacitance so as to limit the modulation response with further RC delay. In view of current high-power devices which are not suitable for communication system, the LD array covered with phosphor film could effectively solve this problem for practical application in the near future. In principle, the light scattering intensity inside a YAG doped dielectric film is proportional to the product of inverse square wavelength and YAG powder size (equivalent to the cube characteristic length). The used phosphor with *x* = (2π*r*)/λ=30π»1 acts as a geometric shape, which would scatter the light according to the projection area. This is opposed to the cases of Mie scattering occurred with *x* ~ 1 and Rayleigh scattering with *x*  1. At such large particle size, the effect of particle size on scattering noise and probability of the incident LD beam is relatively hard to be observed.

**Figure 3:**
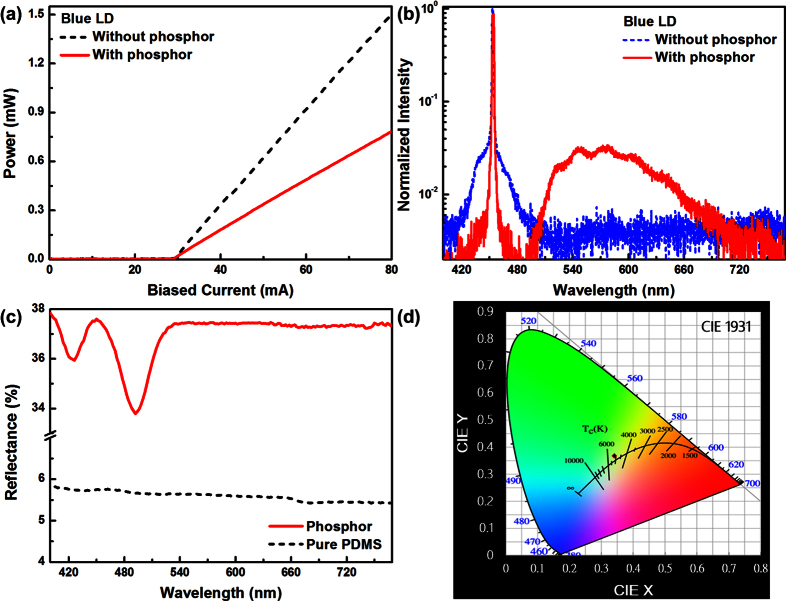
The output characteristics of TO38 packaged blue LD with phosphorous diffuser. (**a**) The power-to-current response of the blue LD with phosphorous diffuser; (**b**) The optical spectrum of the blue LD without or with phosphorous diffuser; **(c)** The reflectance spectrum of the phosphor; **(d)** The chromaticity coordinates result of the yellow phosphorous diffuser film adhered blue LD under CIE 1931.

To verify the effect of phosphorous diffuser film, the power-to-current (*P-I*) response of the blue LD without and with phosphorous diffuser is shown in Fig. 3(a), which is measured by using a visible-light power meter (ILX OMM-6810B + ILX OMH-6732B), and all visible wavelengths are included in the measurement. The threshold current (*I*_*th*_ = 30 mA) keeps invariant without or with the attachment of the phosphorous diffuser film, whereas the slope efficiency (*dP*/*dI)* slightly decreases from 0.9 to 0.82 W/A after adhering the phosphorous diffuser. The reflection at the interface between air and phosphor film has inevitably induced a self-feedback effect, which eventually results in an unwanted injection locking. Furthermore, blue-to-yellow conversion would also cause energy loss for the blue LD beam. These effects decrease the *P-I* slope efficiency of the phosphorous diffuser adhered blue LD. The emission spectrum of the blue LD illuminating phosphor doped diffuser is shown in Fig. 3(b). The reflection spectra of pure and phosphor doped PDMS films with the blue LD are shown in Fig. 3(c). Adding the phosphorous diffuser film inevitably reduces the overall intensity in blue spectral region by 12% due to the emergence of broad yellow emission. To further resolve the white light composed by blue and yellow components from the blue LD and the phosphorous diffuser, the Commission International del1′Eclairage (CIE) chromaticity coordinates were measured by a color analyzer equipped with a CCD detector, as shown in Fig. 3(d). The corresponding chromaticity coordinates (x, y) and correlated color temperature (CCT) falls at (0.34, 0.37) and 5217 K, respectively. Note that the obtained chromaticity is close to the standard CIE (0.3333, 0.3333) of white light. Compared to the results proposed by Denault *et al.*^47^ which show a CCT value of 4400 K with a blue LD excited YAG, the measured CCT value of 5217 K refers to cool white light. Further CCT tuning can be achieved by simply changing different phosphor concentration or mixing different kinds of phosphors.

**Figure 4:**
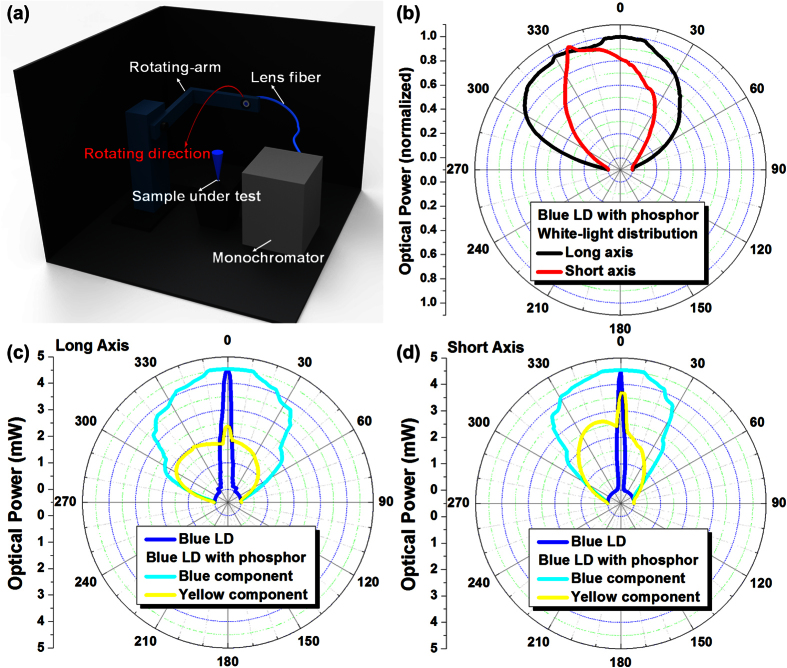
Schematic illustration of light distribution measurement system, and light distribution in polar coordinate of the phosphorous diffuser divergent blue LD (**a**) Angle-dependent light field measurement setup, which consists a rotating-arm, a lens-fiber to collect light, and a monochromator. (**b**) White light distribution of the phosphorous diffuser divergent blue LD in polar coordinate. (**c**,**d**) Light distribution in polar coordinate of blue LD (deep blue curve), phosphor scattered blue (light blue curve) and yellow (yellow curve) luminescent components within the divergent white-light spot. With the radiant angle ranged from −90^o^ to 90^o^, these patterns were taken along long and short axes. Both results show lambertian-like distribution for the divergent white light. For the separated part of blue and yellow light, the distributions show much intense distribution at 0^o^ because of the highly coherent blue light beam, accompanied with divergent lambertian-like distribution at other angles.

Later on, the analysis on bi-directional transmission distribution functions (BTDF) was carried out to estimate the intensity distribution of the phosphorous diffuser diverged blue LD at different emitting angles, as shown in Fig. 4. The measurement of angle dependent optical field distribution is shown in Fig. 4(a). The blue LD light is collected via a fiber by rotating 180^o^ along the plane perpendicular to the output facet of the LD. The position of LD is adjustable to allow its long or short axis aligned on the rotation plane for optical field distribution analysis. The collected light was analyzed by a monochromator, and the blue and yellow components are measured separately at their corresponding dispersion wavelengths. The white-light (including blue and yellow components) distribution is shown in Fig. 4(b), in which the white-light field distribution angle of long axis is wider than that taken along short axis, which results from the asymmetric divergent angle of long and short axes according to the given device geometry. Figures 4(c,d) show the angular radiation pattern of the generated white-light distributions taken along horizontal and vertical axes, respectively. The asymmetric light field is caused by the located position of phosphor film in experiment, which can also be seen from the far-field light distribution of yellow component in Fig. 4(c,d). However, the angle of distributed light field spans from 60^o^ (short axis) to 120^o^ (long axis) after 60-cm propagation in free space. The 0^o^ represents the normal incidence of laser beam on the fiber, whereas the 90^o^ represents a perpendicular angle between fiber and laser beam. As a result, the deep blue curve is a reference showing the blue LD without adding phosphor film, which is only used to present the high directionality of the blue LD in angle-dependent optical field chart. After adding the phosphor diffuser, the optical field of blue component is plotted as the light blue curve with uniformly distributed intensity ranging from −60^o^ to 60^o^. Both distributions show similar lambertian-like shape, contributed by strong scattering effect within the phosphorous diffuser film. In addition, the angle-dependent yellow luminescent components along both axes show high intensity at 0 degree but exhibit lambertian-like distribution at other degrees. The radiation distribution at larger divergent angles makes the phosphor excited yellow light scattered and diverged into lambertian-like distribution.

### The transmission performance of phosphorous diffuser adhered blue LD carried OFDM data over a 60-cm free-space link

**Figure 5:**
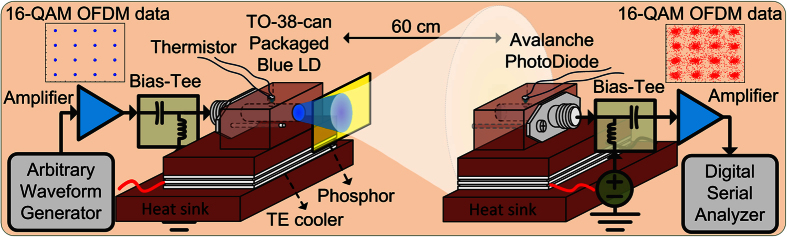
The blue LD with phosphorous diffuser for luminescent white-lighting and optical wireless OFDM communication over a 60-cm free-space link. Experimental setup of phosphorous diffuser diverged blue LD based luminescent white-lighting and 5.2-Gbit/s 16-QAM OFDM data transmission.

For visible light communication test, the experimental setup of the phosphorous diffuser diverged white-light beam from the blue LD with its temperature controlled at 25 °C for both indoor luminescent lighting and wireless 16-QAM OFDM data transmission over a 60-cm free-space link is illustrated in Fig. 5. Since the blue LD is employed as both a lighting source and a data transmitter in the Li-Fi system, its low divergence angle inevitably limits the allowable lighting range although the phosphorous diffuser is adhered. Therefore, the white-lighting intensity of phosphorous diffuser adhered blue LD would correspond to its divergence angle, and it also affects the carried data quality. It means that the transmission performance of proposed white-light source would depend on the direction of the measurement setup which was placed with respect to the laser source. In this work, the phosphorous diffuser adhered blue LD is placed in directly front of the APD receiver with a free-space distance of 60 cm to investigate its maximal allowable transmission capacity. Note that utilizing blue-pass filter could be helpful for suppressing the noise induced by slow yellow light from phosphor. According to the work reported by Sung *et al.*^48^, the OFDM or discrete multi-tone modulation scheme is sensitive to the SNR, and the SNR could inevitably be degraded by applying a blue filter because part of blue light is still blocked by this filter. Only the use of an extremely high blue-transparent filter could further help the transmission performance, but such kind of filter much be designed with special coating or distributed Bragg reflector structure.

**Figure 6:**
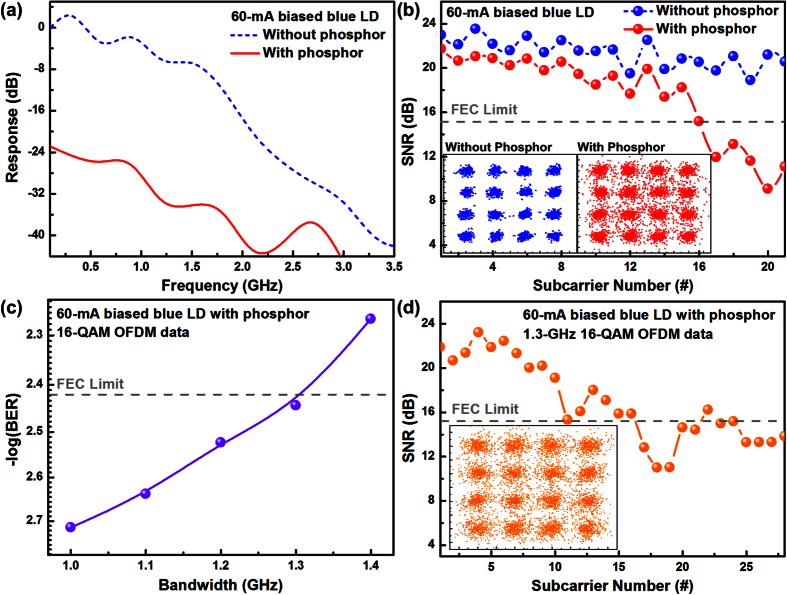
The blue LD with phosphorous diffuser for optical wireless QAM OFDM communication. (**a**) The frequency response of the blue LD without and with phosphorous diffuser. (**b**) The constellation plots and related subcarrier SNRs of the 4-Gbit/s 16-QAM OFDM data carried by the proposed white-light source. (**c**) The BER of phosphorous diffuser adhered blue LD delivered 16-QAM OFDM data at different bandwidths. (**d**) The subcarrier SNRs and related constellation plot of the 5.2-Gbit/s 16-QAM OFDM data carried by the proposed white-light source.

To evaluate the 16-QAM OFDM transmission result when directly modulated by the blue LD after diverging with the phosphorous diffuser, the small-signal analog frequency response of the blue LD biased at 60 mA (~2I_th_) without and with phosphorous diffuser are measured as shown in Fig. 6(a). After applying phosphorous diffuser film for luminescent white-lighting, the throughput modulation response is reduced by around 24 dB because of the relevant absorption, scattering, reflection and beam divergence of the blue laser beam intensity by the phosphorous diffuser film. In addition, both the TO38 laser package and the APD receiver also set a cut-off frequency of modulation at 900 MHz to limit the high-frequency throughout. This confines the allowable OFDM data bandwidth of around 1 GHz for the current VLC system. Subsequently, the 1-GHz 16-QAM OFDM data at 4 Gbit/s is directly encoded to the blue LD without or with adhered phosphorous diffuser film, as shown in Fig. 6(b). Without the phosphor, the delivered data reveals a clear constellation plot with an EVM of 9.3%, whereas the phosphorous diffuser slightly blurs the transmitted constellation plot to increase the EVM up to 15.9%. That is, the phosphorous diffuser helps for spectrally broadening and spatially diverging the blue LD based luminescent lighting; however, such a system still suffers from the degraded SNR by nearly 4 dB (from 21.4 to 17.6 dB) when transmitting the 4-Gbit/s 16-QAM OFDM data due to the significant attenuation on the blue laser beam intensity.

Table 1 summaries the transmission performances of the 4-Gbit/s 16-QAM OFDM data delivered by the phosphorous diffuser diverged blue LD. Although the BER of the received data is degraded from 5.5 × 10^−7^ to 1.8 × 10^−3^ after transferring the linear blue light into a luminescent white light, it can still be decodable by employing a conventional forward error correction (FEC) technique which enables to detect data and correct errors without retransmission^49^,^50^.

Furthermore, to investigate the transmission capacity of the proposed white-light source, the OFDM data bandwidth is increased from 1 to 1.4 GHz, which leads to increase the BER from 2.9 × 10^−3^ to 5.4 × 10^−3^, as shown in Fig. 6(c). Note that a maximum allowable OFDM data bandwidth of 1.3 GHz which represents a raw data rate of up to 5.2 Gbit/s is observed, and a FEC qualified BER of 3.6 × 10^−3^ is also obtained. Moreover, the constellation plot and related subcarrier SNRs of 5.2-Gbit/s data are shown in Fig. 6(d), which represents an average SNR of 15.3 dB and an EVM of 17.3%. Note that the phosphorous diffuser induced throughput degradation on the blue LD output has been compensated by adding the low-noise amplifier (LNA) behind the APD receiver. However, the LNA cannot be unboundedly cascaded as it inevitably induces an extra intensity noise to seriously degrade the SNR of received data. Although the dynamics on data transmission could somewhat be degraded with the aid of phosphorous diffuser, it is the common complaint for all divergent LD sources without using appropriate beam focusing optics. By placing the phosphorous diffuser in front of the blue LD, the phosphor powder absorbs the incident light to enable inherent up transition and then re-emits the luminescence via down transition. Through this process, the data carried by the blue LD fails to distribute over whole white-light spectrum owing to the slow spontaneous emission process of the phosphor, which cannot be transferred to other luminescent bands of the white-light beam as the yellow light could not be modulated as quickly as the LD. That is, the phosphor induced broadband luminescence is useful for white-lighting but a source of noise in the VLC system. In this case, a perfect photodiode should be set to detect only the blue light to avoid the SNR degradation by white-light source. Moreover, the transmission results of proposed white-light source is independent from the light polarization of blue LD since the phosphorous diffuser transfers the stimulated emitting photons of blue laser beam into the spontaneous emitting photons of proposed white-light beam. The blue LD based Li-Fi system has already shown white-lighting feature and somewhat better wireless data streaming when comparing with currently available LED based white-lighting system covering free-space wireless link. Nevertheless, the impressed experimental result is that wouldn’t be an issue to limit its transmission performance while offering a certain angular range of illumination, and the dual functionalities of the LD based Li-Fi system is emphatically the proper and indispensable intention for extending the applicability of the GaN based blue LDs in next-generation lighting industry. Extensive capabilities on network data formats are easy and intuitive as well, although trawling through the access of different contents of data-streams is unavailable to be performed in this work. Such impressive design is adaptable with various luminescent phosphorous diffuser at any desired colors, which endows with all available key processing and decoding options, and is still plenty for versatile transmission formats to suit practical needs of current LED based lighting system.

## Conclusion

In this work, a phosphorous diffuser diverged blue GaN LD is successfully demonstrated for both indoor luminescent white-lighting and optical wireless communication over a 60-cm free-space link, which represents a current state-of-the-art performance for carrying 16-QAM OFDM data at 5.2 Gbit/s. The luminescent phosphor doped diffuser diverges the blue laser beam to a white-light spot covering a radiant angle of 120 degree with CIE coordinates of (0.34, 0.37) for indoor lighting. The diverged blue LD beam degrades its throughput response by 24 dB due to the high reflection/scattering/absorption of the blue laser beam when passing through the phosphorous diffuser film. Without the phosphorous diffuser, the delivered 4-Gbit/s 16-QAM OFDM data reveals a clear constellation plot with an EVM of 9.3%, an average SNR of 21.4 and a BER of 5.5 × 10^−7^. The phosphorous diffuser diverged luminescent light spot degrades the EVM to 15.9%, the average SNR to 17.6 dB and the BER to 1.8 × 10^−3^. By further increasing the OFDM data bandwidth to 1.3 GHz, the proposed white-light source still exhibits its ability to carry the 5.2-Gbit/s 16-QAM OFDM data with an EVM of 17.3%, an SNR of 15.3 dB and a BER of 3.6 × 10^−3^. Versatile application choices allow the blue LD based Li-Fi system with up-scaling capability on communication and lighting aspects as an alternative approach. This report has declared the capability of the phosphorous diffuser diverged GaN blue LD as being a potential candidate in next-generation indoor lighting and communication industries.

## Methods

### Fabrication of phosphorous diffuser

To transfer the blue laser beam into the white-light luminescence, the adhered phosphorous diffuser film was fabricated by spin-coating the poly-dimethylsiloxane (PDMS) pre-polymer based aqueous solution with doped phosphorous powders. The PDMS-suspension is included due to the high stability and high transparency in the visible light region. The yellow phosphor used in this experiment is YAG with particle size of 13 μm. The concentration of the phosphor in the PDMS solution used to form the phosphor-suspension slurry is about 12%. Subsequently, the phosphor-PDMS solution was uniformly dispensed onto the glass substrate and spin-coated at 700 rpm for 60 s, then baked at 70 °C for 2 hours. The thickness and concentration of fabricated phosphor diffuser can lead to significant influence on both white-lighting and data transmission qualities. The dense phosphor causes the decrease of CCT and degradation of data transmission rate (due to low signal-to-noise ratio). In contrast, the insufficient phosphor concentration results in a non-scattered laser beam, which is harmful for human eyes. In this case, the phosphor concentration and fabrication parameters are optimized to simultaneously acquire high-quality white-lighting and high-speed transmission performances. Finally, the phosphorous diffuser film was peeled from the glass substrate and transferred to a mount as a free-standing phosphor film for adhering at the output window of the blue GaN LD.

### Analytic setup for luminescent white-lighting and optical wireless communication

In the testing bench, the electrical 16-QAM OFDM data with a FFT size of 512, a cyclic prefix of 1/32, a subcarrier number of 28 and a covering bandwidth of 1.3 GHz was generated by a homemade MATLAB program. To represent the 16-QAM OFDM data at a raw data rate of 5.2 Gbit/s, the generated waveform was uploaded into an arbitrary waveform generator (Tektronix, 70001A) with a sampling rate of 24 GSa/s. After power-level amplification with 10-dB gain in a ultra-broadband amplifier (Picosecond Pulse Labs, 5828), the pre-amplified 16-QAM OFDM data was combined with a DC bias current via a bias tee (Mini-Circuit, ZX85-12G-S+) to concurrently drive and encode the blue LD (OSRAM Opto Semiconductors, PL 450B). In this work, the blue LD is biased at 60 mA to approach a balance between the relative intensity noise suppression and the throughput intensity declination. The yellow phosphor was put in front of the blue LD as a divergent luminescent diffuser, which provides divergent white-light beam for both indoor luminescent white-lighting and 16-QAM OFDM data transmission. After free-space propagation, the diffused white-light beam with carried data was launched into an avalanche photodiode receiver (APD, Hamamatsu, S9073) with a 3-dB bandwidth of 900 MHz. After receiving, the data was further amplified by a LNA (Mini-Circuit, ZKL-1R5) with 40-dB gain, and its waveform was captured by a digital serial analyzer (Tektronix, DSA71604C) with a resolution of 100 GSa/s for decoding and analyzing its constellation plot, EVM, SNR and BER.

## Additional Information

**How to cite this article**: Chi, Y.-C. *et al.* Phosphorous Diffuser Diverged Blue Laser Diode for Indoor Lighting and Communication. *Sci. Rep.* **5**, 18690; doi: 10.1038/srep18690 (2015).

## Figures and Tables

**Figure 1:**
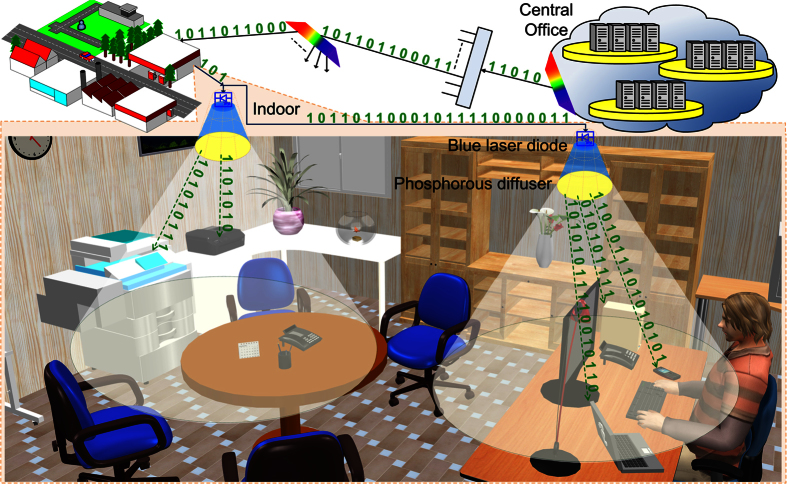
The schematic diagram of phosphorous diffuser diverged blue LD for indoor white-lighting and optical wireless communication. Versatile application choices allow the blue LD based Li-Fi system with up-scaling capability on communication and lighting aspects. *Sweet Home 3D, Copyright (c) 2005–2015 Emmanuel PUYBARET/eTeks <*info@eteks.com*>.*

**Table 1:** The summarized transmission parameters of the 4-Gbit/s 16-QAM OFDM data carried by blue LD without and with phosphorous diffuser.

	EVM	SNR	BER
Without phosphor	9.3%	21.4 dB	5.5 × 10^−7^
With phosphor	15.9%	17.6 dB	1.8 × 10^−3^
